# Timing of Treatment for Patients with Hypertrophic Maxillary Labial Frena

**DOI:** 10.3390/dj13090414

**Published:** 2025-09-08

**Authors:** Veronica Lexa Marr, Lauren Grace Stewart, Man Hung, Val Joseph Cheever

**Affiliations:** College of Dental Medicine, Roseman University of Health Sciences, 10894 S. River Front Parkway, South Jordan, UT 84095, USA; lstewart482@student.roseman.edu (L.G.S.); mhung@roseman.edu (M.H.); jcheever@roseman.edu (V.J.C.)

**Keywords:** frenum, frenulum, frenectomy, maxillary labial frenum, superior labial frenum, tethered frenum, hypertrophic frenum, midline diastema

## Abstract

**Background/Objectives**: The maxillary labial frenum (MLF) is a connective tissue structure attaching the upper lip to the maxillary alveolar process. Its morphology varies significantly among individuals and is often most prominent during early childhood. While hypertrophic or low-attaching frena have been associated with diastemas, feeding issues, and speech impairments, there is no causal evidence supporting early surgical intervention. This review aims to examine current evidence regarding the timing and necessity of frenectomy procedures and to evaluate the implications of early versus delayed intervention. **Methods**: A narrative review was conducted using twenty peer-reviewed articles published in the past 10 years, with one additional article from 2012 included for its ongoing relevance. Databases searched included PubMed, the NIH database, the Reference Manual of Pediatric Dentistry, and journals from the American Academy of Pediatrics. Inclusion criteria were English-language, peer-reviewed studies that addressed the morphology, classification, diagnosis, management, and outcomes of MLFs across age groups. Excluded were studies focusing solely on mandibular, buccal, or lingual frena; non-English publications; case reports; and articles lacking full-text availability. **Results**: The literature suggests that premature frenectomy, prior to the eruption of permanent maxillary canines, typically between 9 and 12 years of age, is associated with frenum regrowth, surgical complications, and orthodontic relapse. Additionally, a lack of standardized diagnostic criteria contributes to inconsistent clinical decision-making. Conservative management, including monitoring, is strongly recommended as the frenum often migrates apically as the maxilla develops. Factors such as airway obstruction and developmental delays should be ruled out before considering surgery. **Conclusions**: There is insufficient evidence to support early surgical intervention for MLF-related concerns. A conservative, individualized approach, delaying frenectomy until after permanent canine eruption, may minimize complications, improve long-term outcomes, and allow the frenum to migrate apically as the patient develops. Standardized diagnostic tools are urgently needed to guide clinical decision-making.

## 1. Introduction

In the oral cavity, there are seven distinct frena. These include the maxillary labial frenum (MLF), four buccal frena, a lingual frenum, and a mandibular labial frenum [1,2]. The MLF is a mucous membrane fold that connects the buccal surface of the upper lip to the alveolar mucosa, gingiva, and periosteum near the maxillary central incisors. The MLF consists of dense connective tissue containing collagen and elastic fibers but lacks muscle fibers [3]. Its primary function is to stabilize the upper lip [2,4,5].

Variations in the MLF are believed to be associated with clinical, functional, and aesthetic concerns, such as latching, speech, gingival recession, and diastema [6]. Abnormally prominent or attached frena may attach to the interdental papilla or further, exerting tension on the gingiva [1,3,5]. This pulling force can prevent the natural converging of the central incisors during orofacial and skeletal development. This space is referred to as diastema. 

Historically, frenectomies have been performed to address the concerns. However, research suggests that premature intervention may lead to unnecessary surgical risks, including scarring, postoperative complications, and orthodontic relapse [7,8]. Delayed intervention until the eruption of the permanent maxillary canines may provide more predictable outcomes by allowing for natural diastema closure. This can minimize the need for surgical correction. Despite these findings, clinical approaches to MLF management remain inconsistent. Understanding optimal timing for intervention and establishing a consistent approach to management is necessary to improve patient outcomes.

The initial search across databases yielded 51 articles. Of note, 11 duplicates were removed, and the remaining 40 records were screened by 4 independent reviewers. Following this, 7 reports were not retrieved, and the remaining 35 reports were assessed by reviewers for eligibility. Of these remaining records, nine were focused on the other six frena, not the maxillary labial area. Two were exclusively focused on surgical technique, which did not address the research topic of determining the timing of intervention for MLF only. Two additional articles were non-English texts and were subsequently removed. Consequently, 20 remaining studies met all inclusion criteria.

## 2. Materials and Methods

A narrative review was conducted to evaluate the morphology, classification, and clinical implications of the MLF. This search utilizes twenty peer-reviewed articles published in PubMed, the National Institute of Health (NIH) database, the Reference Manual of Pediatric Dentistry, and the American Academy of Pediatrics, among other journals. 

Inclusion criteria for this review consisted of peer-reviewed, English-language studies published within the last 10 years, except for one study from 2012, included due to its continued relevance to the topic. Selected studies specifically focused on the MLF, including its clinical characteristics, diagnosis, management, and outcomes across different age groups. Exclusion criteria included articles referencing mandibular labial frena, buccal frena, or lingual frena only. Journals with non-English texts, regarding individual case reports, and studies without full-text availability were also excluded. 

Two independent reviewers conducted the journal selection process. First, titles and abstracts were screened for relevance. Then, full-text articles meeting the initial criteria were retrieved and assessed for inclusion based on the criteria. Discrepancies in study selection were resolved through discussion or consultation with a third reviewer. A fourth reviewer completed an overall analysis of the articles included to ensure consistency, relevance, and alignment with the research objective.

The terms tethered, hypertrophic, and restrictive MLF are often used interchangeably in clinical settings, yet they refer to distinct anatomical and functional variations. A hypertrophic frenum refers primarily to an increase in tissue volume or thickness, often without associated limitation of movement or function [9]. A tethered frenum, by contrast, implies an abnormal attachment or positioning [10]. This could include extension into the interdental papilla or palatal tissue that can interfere with normal oral functions or contribute to diastema formation [1,5]. The term restrictive is typically reserved for cases in which the frenum physically limits the range of lip or oral movement, often contributing to feeding difficulties, speech impairment, or orthodontic challenges [1]. All three presentations will be discussed collectively, as the timing of intervention, treatment options, and associated clinical considerations are largely consistent across these classifications.

## 3. Results

This discussion synthesizes the current literature on hypertrophic MLFs, focusing on treatment timing, clinical relevance, and orthodontic considerations.

The timing of treatment for hypertrophic MLF is of ongoing debate, influenced by age, functional impairments, and orthodontic considerations. Current evidence supports a conservative approach, suggesting a delayed intervention until natural skeletal and odontogenic developmental changes have occurred. In rare and severe cases, significant clinical issues may necessitate earlier action.

In most individuals, the MLF undergoes natural regression with growth, migrating apically during maxillary development. This regression reduces its prominence and, in many cases, resolves associated diastemas without a frenectomy. Some studies indicate that surgical procedures during infancy or early childhood are rarely warranted, as abnormalities often self-correct with growth [5].

In cases where feeding difficulties arise in infants and young children, clinicians are advised to eliminate other potential causes, such as nasal or airway obstruction, before considering frenectomy [8]. Research indicates no direct correlation between frenum attachment and breastfeeding problems, further supporting a conservative approach [10].

Studies recommend postponing frenectomy until after the eruption of permanent maxillary canines. Eruption occurs between 9 and 12 years old, with some variability. Postponing treatment allows for the natural resolution of diastemas and avoids premature surgical intervention that might lead to orthodontic relapse due to post-surgical scarring [1,5].

### 3.1. MLF Misconceptions of Associated Issues

As children grow, the vertical development of the alveolar process and eruption of permanent teeth often cause an enlarged frenum to recede and lose prominence. This growth can resolve diastema without intervention [5,11].

Although some articles suggest that anatomical variations in the frenum may contribute to issues such as speech difficulties, chewing problems, and challenges with infant latching, several reports have found no significant evidence indicating that hypertrophic MLF attachments cause these impairments [6,11,12]. Observational studies and systematic reviews repeatedly report that no direct or consistent association has been found between MLF abnormalities and difficulties with breastfeeding or speech development in most cases [12]. Consistent with this, a recent cross-sectional cohort study reported no correlation between MLF attachment and breastfeeding success in infants. This further supports that hypertrophic MLF appearance alone is not predictive of the functional abilities of a patient [6].

By contrast, a small case-series report described infants with an isolated enlarged MLF who received surgical intervention between 1 to 12 weeks old. All 7 infants experienced improved breastfeeding and weight gain following their frenectomies [13]. While these findings suggest that an enlarged frenum may contribute to breastfeeding difficulties in select cases, the evidence is limited by very small sample sizes, retrospective design, and lack of controls. Additionally, outcomes were parent-reported instead of a standardized assessment. Lacking a comparison group prohibits an analysis of a patient’s natural improvement or the potential of a placebo effect. Case series are also unable to establish causation. Larger trials with standardized measures of outcomes compared to a control group are needed to determine a causal effect.

Evidence from a prospective blinded cohort study of 100 newborns found no correlation between the classification of the maxillary frenulum and breastfeeding success. Patients’ frena were assessed using both the Kotlow and Stanford classification systems. Despite the subjective nature of these metrics, subjects’ MLF attachment indicated no significant difference in maternal pain scores or latch quality [14]. The findings indicated that anatomy alone is insufficient to predict breastfeeding outcomes.

### 3.2. Determining Irregularity

Determining whether a MLF is irregular involves assessing its attachment, morphology, and associated functional concerns. Clinically, an abnormal frenum is characterized by a hypertrophic attachment that extends into the interdental papilla or, more severely, to the palatal gingiva between the incisors. This is identified with the blanching test, where upward movement of the upper lip causes a visible blanching of the attached gingiva, indicating a restrictive frenum [5]. Current diagnostic criteria emphasize the need to differentiate between a naturally prominent but non-restrictive frenum and one causing significant functional or aesthetic concerns [7].

In clinical and pediatric dentistry, the Kotlow classification system and standard classification system are used to categorize MLF attachments. The Kotlow classification system is based on the frenum’s insertion point relative to the alveolar ridge, from the lowest grade of frena having minimal alveolar mucosa attachment, and with the highest grade indicating an attachment extending into the anterior papilla and the hard palate [15,16]. While its anatomical simplicity makes it easy to apply, recent research has questioned its accuracy and clinical relevance. The system fails to account for the natural growth and apical migration of the frenum during childhood development, leading it to label many infants with a degree of restriction [15]. Additionally, the Kotlow rating system has been shown to be difficult to reproduce, given its poor interrater reliability [15].

The Stanford classification system instead categorizes MLFs into three different types based on the single anatomical parameter of the point of attachment of the MLF [16]. This system has shown slightly more consistent interrater reliability as it combines Kotlow grades into the middle of its grading scale. Both the Kotlow and Stanford Classification Systems often overemphasize visual grading, leading to the overdiagnosis of lip-ties in infants without functional impairments. This system can result in unnecessary surgical referrals, as most infants with visible ties do not experience feeding or speech difficulties [15,16]. For future clinical decisions to be sound, a new diagnostic tool is needed to integrate both structural extensivity and functional impairments, rather than depending solely on positional anatomy.

More recent studies have attempted to refine these classification systems to improve diagnostic accuracy and clinical relevance. A 2025 cross-sectional study analyzed the prevalence of attachment and the morphological variations between frena in their sample. While the type of area, mucosal, gingival, papillary, and papilla penetrating remains similar to the previously relied-on methods of classification, the addition of the morphology and comparison of age and gender within the study induced more robust results. Their most common finding of children with a simple and gingival type frenum demonstrates less exaggerated results than when the Stanford or Kotlow Classification Systems are applied [17]. Similarly, a 2022 article compared the Kotlow and Stanford systems with their HOP-ROC scale, including more specific parameters, including the thickness of the gingival and labial attachment of the frenum, the length of frenum stretch and from the alveolar edge to the gingival attachment, and the free-lip to total-lip ratio. This enhanced scale provided significantly improved interrater results [16]. It is critical that future studies use classification systems that account for the rapid changes in the position of the frena based on the age and development of the patient.

### 3.3. When to Treat Early

In very rare and severe cases, early surgical intervention may be required depending on the severity of the condition and the impact on the patient’s oral and functional health. Evidence suggests that diastemas wider than 2 mm are unlikely to close spontaneously, supporting early frenectomies [1]. The renum attachment is a primary indicator of early intervention.

If it extends deeply into the interdental papilla or palatal mucosa and exerts extensive tension, it may hinder the effectiveness of future orthodontic treatment, eliminating the benefit of delayed intervention, as it is unlikely to close spontaneously [5].

Early intervention may be necessary for functional impairments such as speech issues, oral hygiene challenges, or gingival recession if other possible contributing factors have been ruled out [6,10].

Psychosocial concerns regarding persistent diastema rarely justify early treatment, as natural apical recession is preferred cosmetically to an initial incision with the additional risk of further surgical intervention due to high recurrence rates. If there are no significant functional or psychosocial concerns, and the diastema measures less than 2 mm, intervention is unnecessary until the eruption of permanent maxillary canines [1].

A comprehensive clinical and radiographic evaluation in collaboration with an orthodontist or pediatric dentist ensures that the timing and approach to treatment are tailored to the individual patient’s needs.

### 3.4. Potential Problems When Treating Too Early

Treating a MLF before intervention is necessary can lead to complications. Early frenectomies, particularly in young children, may cause scarring that restricts adjacent soft tissues and interferes with natural oral development.

Premature surgical intervention is especially problematic when performed before dental and skeletal growth has concluded. The maxilla undergoes significant growth anteriorly and coronally throughout childhood. Early treatment may impede the natural developmental processes regarding spontaneous diastema closure and disruption of subsequent orthodontic treatment outcomes [1].

Infants and young children are particularly vulnerable to surgical complications due to their underdeveloped immune systems, lower platelet counts, and fragile tissues. These factors can result in delayed healing and higher susceptibility to infections. It typically takes several months to a few years for immunoglobulin levels and platelet counts to reach levels sufficient for minimizing surgical risks [18].

Concerns regarding feeding difficulties are often cited to justify early intervention. However, research suggests that breastfeeding problems are rarely attributed solely to frenum restrictions and are more likely related to other anatomical or functional issues [10]. Routine surgical intervention during infancy or early childhood is generally unwarranted unless severe functional impairments are present. Deferring treatment until skeletal and dental development can show a clearer picture of the MLF. It provides more predictable outcomes and minimizes the risk of complications, allowing optimal dental and functional development [1,18].

### 3.5. How to Treat

Observation is appropriate in most cases of restrictive MLF. For rare and severe cases, there are two main surgical options for patients with severe MLF restriction that impedes function beyond a reasonable point. A frenectomy includes a simple release of the frenum to eliminate the restriction of the upper lip. A frenuloplasty or frenulectomy will reposition the frenum in addition to releasing the restriction. A 2023 systematic review presented treatment options regarding a scalpel or laser approach. For optimal treatment, laser-assisted frenectomies are preferred due to their precision, reduced bleeding, and faster recovery time [3,19].

### 3.6. MLF Relationship to Diastemas

Prominent or low-attaching frena are often associated with diastemas due to tension exerted on the interdental papilla [3]. However, studies suggest that not all individuals with a pronounced MLF develop diastema, and not all diastemas are caused by an abnormal frenum [7,19]. Diastemas may arise from other factors. These include variations in tooth size, abnormal dental arch growth, or habits like thumb sucking [20]. For many individuals, diastemas resolve spontaneously as the frenum migrates apically during growth and the permanent maxillary canines erupt. Therefore, routine surgical treatment is unwarranted unless the diastema fails to close naturally or is associated with residual functional impairments after the permanent maxillary canines are fully erupted. A multidisciplinary approach involving orthodontists and pediatric dental specialists is often the best course of action for managing such cases [1,5].

### 3.7. Orthodontics and MLF Treatment

In orthodontic contexts, and in rare and severe cases where the MLF does not self-correct, a frenectomy should be performed after closure of the diastema during orthodontic treatment. This timing will help prevent relapse caused by residual tension on the gingiva. Premature intervention can disrupt orthodontic outcomes, highlighting the importance of aligning surgical timing with comprehensive orthodontic planning [9].

### 3.8. Lack of Caries and Hypertrophic Frenum Association

There is a lack of significant correlation between the presence of an enlarged MLF and the development of dental caries. No statistically significant differences were observed when comparing affected individuals to the control group. These findings further challenge the necessity of routine early surgical intervention for an MLF. However, maintaining good oral hygiene is pertinent to said results [10].

### 3.9. Limitations

This review relies on an analysis of the existing literature rather than original clinical trials, limiting the ability to control variability in study design, sample populations, and methodologies. As a result, differences in patient selection, diagnostic criteria, and treatment protocols across sources may introduce inconsistencies in the reported outcomes. 

This analysis focuses solely on the MLF without considering the potential impact of other aberrant frenal attachments. Patients with multiple enlarged frena may have different treatment needs.

This study cannot account for all individual patient factors that may influence clinical decision-making. Genetic predisposition, oral habits, or variations in skeletal growth patterns need to be determined on a case-by-case basis. Most existing studies emphasize short-to-mid-term outcomes. This leaves understudied long-term data pertaining to relapse rates, periodontal health, and functional changes post-frenectomy. While psychosocial considerations such as patient or parental concerns about esthetics are acknowledged, their subjective nature makes it difficult to quantify and standardize across studies. Future research, including controlled longitudinal studies, is necessary to refine treatment guidelines and establish a more standardized approach to MLF management.

## 4. Discussion

The studies reviewed consistently highlight the importance of timing in the treatment of hypertrophic MLF, with a strong consensus supporting the postponement of frenectomy until after the eruption of permanent maxillary canines, typically between the ages of 9 and 12 years. This recommendation aligns with findings that the MLF naturally regresses during growth, often leading to the spontaneous closure of associated diastemas without the need for surgical intervention.

**Figure 1:**
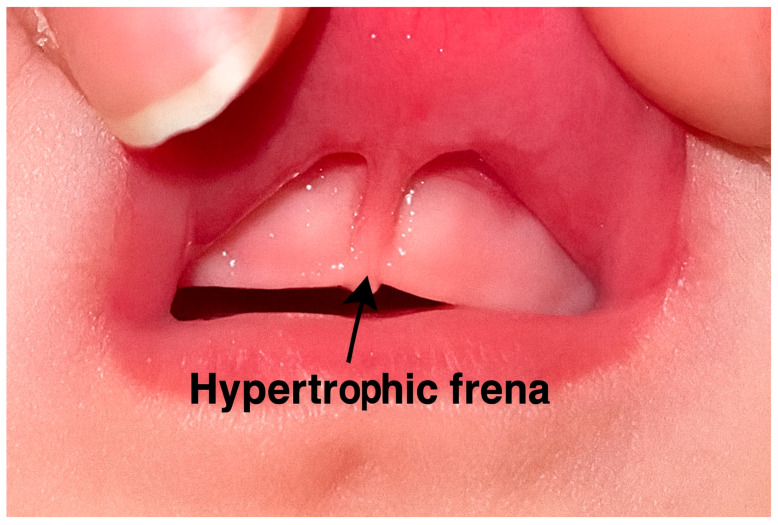
Maxillary labial frenum attachment in early childhood; clinical presentation of a tethered maxillary labial frenum in an infant patient.

Figure 1 illustrates an infant patient with a maxillary labial frenum that is visibly tethered. It attaches low on the alveolar ridge and extends toward or into the interdental papilla between the developing primary central incisors. This hypertrophic frenum is commonly thought to interfere with oral functions such as latching during breastfeeding and speech development. However, current evidence shows no statistically significant correlation between surgical intervention and long-term improvement of these functional issues [10]. Additionally, there is no established link between a tethered maxillary labial frenum and oral hygiene complications [10]. Given the potential for severe complications associated with invasive procedures in early childhood, such as scarring, delayed healing, and interference with normal growth, clinicians are advised to approach early surgical intervention with caution.

Table 1 presents a summary of results compiled from multiple studies demonstrating the timing of MLF intervention. The table outlines the likelihood of natural diastema closure, orthodontic relapse, and complication rates based on age at the time of treatment [5,9,20]. In infancy and early childhood, there is a high likelihood of natural diastema resolution, though high orthodontic relapse rates. In contrast, interventions performed during the mixed dentition and adolescent phases are associated with a lower risk, fewer complications. There are predictable healing times due to stabilized growth.

Table 2 shows the results from a prospective observational cohort study comparing 61 infants with a tethered MLF (study group) and 66 infants with no abnormal frena attachment (control group). The groups were observed over a period of 6.42 years. This table compares dental indicators and treatments between patients with an extended maxillary labial frenum (study group) and those without (control group). No statistically significant differences were observed between the study and control groups in the prevalence of tooth decay, number of fillings, or extractions over the past two years [10]. These findings suggest that the presence of an extended MLF is not independently associated with increased dental pathology and is not reason enough to provide surgical intervention.

In orthodontic practice, the importance of waiting for permanent teeth to erupt before performing a frenectomy is highlighted. Premature intervention can disrupt orthodontic outcomes by causing scarring or relapse due to the residual tension exerted by the frenum. Moreover, in cases where the MLF does not self-correct and causes significant functional impairments, surgical intervention should be considered after the permanent canines have erupted to minimize complications.

The analysis also finds a lack of evidence for a direct link between MLF abnormalities and significant functional problems, such as speech or feeding difficulties, which often resolve naturally with growth. When functional impairments do arise, other potential causes, such as airway obstructions, must be ruled out before considering frenectomy [20].

Future research on hypertrophic MLF abnormalities should focus on developing standardized, validated tools for assessing and grading frenum variations. The lack of a universally accepted classification system complicates both clinical decision-making and the ability to compare outcomes across studies. A reliable grading scale could enhance diagnostic consistency and improve the evaluation of treatment efficacy.

Research is often limited by ethical considerations, particularly in pediatric populations. The relatively low prevalence of clinically significant MLF abnormalities makes it challenging to conduct large-scale studies with adequate control groups [12].

Another critical area for advancement is the coordination of research with related conditions, such as tongue-tie, where interventions may overlap. Studies should aim to separate the effects of MLF abnormalities from other orofacial conditions to provide clearer guidance for treatment. Finally, professional organizations like the American Academy of Pediatric Dentistry and the American Academy of Pediatrics should collaborate to produce a joint statement on the evaluation and management of MLF. A consensus regarding the timing of treatment would reduce variability in clinical practice as well as guide future research questions evolving in this field.

## 5. Conclusions

The optimal timing for hypertrophic MLF treatment is after the eruption of the permanent maxillary canines, usually between the ages of 9 and 12. Waiting for these developmental milestones aligns treatment with the natural regression of the MLF, allowing for spontaneous or orthodontically guided diastema closure. This approach of delaying treatment significantly reduces the need for invasive interventions.

It is critical to exhaust all other treatment options and thoroughly evaluate alternative causes for functional impairments before MLF intervention. Early surgical interventions performed prior to significant dental and skeletal growth carry increased risks [5].

Although rare, severe functional impairments such as feeding difficulties or speech impairments may necessitate earlier treatment. Such cases should be individually and carefully evaluated. Pediatric dental specialists emphasize that alternative causes for these problems, including airway obstructions, should be ruled out before considering frenectomy [10,20]. The use of laser-assisted frenectomy has further optimized outcomes, allowing for precise and minimally invasive procedures [19].

Delaying frenectomies until after orthodontic intervention can ensure that the diastema is closed and retained without gingival tension from the frenum. This approach minimizes complications and aligns with the developmental milestones of the oral cavity to provide the most stable and predictable outcomes for patients [1]. Thus, surgical intervention should occur after the eruption of maxillary canines for optimal dental and functional development.

## Figures and Tables

**Table 1:** Age-Related Clinical Outcomes Following Maxillary Labial Frenum Treatment [1,5,8,9,20].

Age of Intervention	Likelihood of Natural Diastema Resolution	Orthodontic Relapse Rate	Complication Rate
0–8 years	High [5,20]	High [1,5]	High [8]
9–12 years	Low [1,8]	Low [8]	Low [8,9]
13+ years	Low [5,20]	Low [9]	Low [9]

Interventions performed during mixed dentition and adolescent phases are associated with lower risk, fewer complications, and predictable healing [1,5,8,9,20].

**Table 2:** Comparison of Dental Problems and Treatments in Patients with and without an Extended Maxillary Frenum [10].

Dental Problems and Treatment	Control	Study	*p*-Value
Pt. suffering from tooth decay	1.4 ± 1.6	0.9 ± 1.7	0.585
Pt. has undergone fillings in the last 2 years	30 (45.5)	26 (42.6)	0.858
Pt. has undergone extraction in the last 2 years	7 (6)	5 (8.2)	0.765

The lack of statistically significant findings between frenum attachment groups suggests that the presence of an extended MLF is not independently associated with increased dental pathology [10].

## Data Availability

The original contributions presented in this study are included in the article. Further inquiries can be directed to the corresponding author.

## Abbreviations

The following abbreviations are used in this manuscript:

MLF	Maxillary labial frenum
